# Rapid Detection of *Bacillus anthracis* Spores Using Immunomagnetic Separation and Amperometry

**DOI:** 10.3390/bios6040061

**Published:** 2016-12-20

**Authors:** David F. Waller, Brian E. Hew, Charlie Holdaway, Michael Jen, Gabriel D. Peckham

**Affiliations:** 1 Black Ivory Biotech, Inc., P.O. Box 893128, Mililani, HI 96789, USA; dfw808@yahoo.com (D.F.W.); hewbrian@gmail.com (B.E.H.); 2 Holatron Systems, LLC., 833 Ilaniwai Street, Suite 2, Honolulu, HI 96813, USA; charlie@holatron.com; 3 Mike Jen Software Services, 1035 Aster Ave #2103, Sunnyvale, CA 94086, USA; mike.jen@gmail.com

**Keywords:** bioterrorism, amperometry, anthrax spores, biodetection, immunoassay, portable assay

## Abstract

Portable detection and quantitation methods for *Bacillus anthracis* (anthrax) spores in pure culture or in environmental samples are lacking. Here, an amperometric immunoassay has been developed utilizing immunomagnetic separation to capture the spores and remove potential interferents from test samples followed by amperometric measurement on a field-portable instrument. Antibody-conjugated magnetic beads and antibody-conjugated glucose oxidase were used in a sandwich format for the capture and detection of target spores. Glucose oxidase activity of spore pellets was measured indirectly via amperometry by applying a bias voltage after incubation with glucose, horseradish peroxidase, and the electron mediator 2,2′-azino-bis (3-ethylbenzthiazoline-6-sulphonic acid). Target capture was mediated by polyclonal antisera, whereas monoclonal antibodies were used for signal generation. This strategy maximized sensitivity (500 target spores, 5000 cfu/mL), while also providing a good specificity for *Bacillus anthracis* spores. Minimal signal deviation occurs in the presence of environmental interferents including soil and modified pH conditions, demonstrating the strengths of immunomagnetic separation. The simultaneous incubation of capture and detection antibodies and rapid substrate development (5 min) result in short sample-to-signal times (less than an hour). With attributes comparable or exceeding that of ELISA and LFDs, amperometry is a low-cost, low-weight, and practical method for detecting anthrax spores in the field.

## 1. Introduction

The potential threat of anthrax spores being used as a biological weapon calls for a quick and reliable means of field detection that is portable, cost-effective, and easy to operate. Current field-deployable methods of anthrax spore detection such as lateral-flow devices (LFDs) lack sensitivity and quantitative ability, and are subjectively interpreted [1,2,3]. More sensitive detection methods such as ELISA or PCR typically use time-consuming protocols and instruments not suitable for field use or require sample enrichment or clean-up prior to testing to avoid inhibition by environmental contaminants [4,5,6,7].

Immunomagnetic separation (IMS) is a technique in which antibodies specific to a target organism are immobilized on magnetic beads to enable fast and efficient concentration and purification of the target from crude samples, thus reducing or eliminating any matrix effects from contaminants in subsequent testing. IMS has been used previously in combination with other techniques for the detection of *Bacillus anthracis* spores in various matrices [8,9,10,11,12]. The large surface area available on magnetic beads for the immobilization of antibodies allows for an efficient and sensitive capture of spores, although the binding specificity and limit of detection generally depend on the antibodies and binding conditions used. In this study, IMS is combined with amperometry, which offers the advantages of being very sensitive, robust, and economical and can be made compact and easy to use in the field [13].

The method presented here uses 2,2′-azino-bis (3-ethylbenzthiazoline-6-sulphonic acid) (ABTS) as a soluble redox mediator in the presence of glucose and horseradish peroxidase (HRP) for the indirect measurement of glucose oxidase activity on IMS-captured anthrax spores detected with monoclonal antibody-glucose oxidase (GOX) conjugate. The oxidation of ABTS by HRP using hydrogen peroxide produced in the reaction of glucose oxidase with glucose generates a stable, green-colored ABTS cation radical by loss of an electron [14], a reaction that is commonly used in colorimetric assays [15]. Amperometric measurement of ABTS oxidation by laccase and other multicopper oxidases has been described previously [16,17,18,19,20]; however, to our knowledge, the use of ABTS as a soluble amperometric mediator in glucose oxidase-based detection systems with HRP has not been reported before. With this strategy, a threefold greater signal could be generated in 5 min compared to 30 min for the soluble redox mediator 2,6-dichlorophenolindophenol (DCPIP) [21]. This allows for shorter run times, an important consideration for assays designed for use in the field. To that end, in this work, we demonstrate the performance of the assay in pure culture as well as in the presence of various possible environmental interferents including soil that could affect the testing of actual field samples.

## 2. Materials and Methods

All reagents, supplies, and equipment were purchased from VWR (Radnor, PA, USA) unless otherwise indicated. 2,2′-Azinobis(3-ethylbenzothiazoline-6-sulfonic acid) ammonium salt (ABTS) was from TCI (Tokyo, Japan, cat# A2166). Horseradish peroxidase (150–200 units/mg solid) was from MP Biomedicals (Solon, OH, USA, cat# 195372). Bovine serum albumin (BSA) was from Fisher Scientific (Pittsburgh, PA, USA, cat# BP1605-100). D-Glucose was from Sigma-Aldrich (St. Louis, MO, USA, cat# G5767). Potting soil samples were purchased from City Mill (Honolulu, HI, USA).

### 2.1. Spore Production

All *Bacillus* strains used in this research were obtained from BEI Resources (Manassas, VA, USA) including the *B. anthracis* Sterne strain (cat# NR-1400), *B. thuringiensis* (NR-610), *B. cereus* (NR-608), and *B. mycoides* (NR-612). Each strain was inoculated into 5 mL of LB broth (Difco 244620, Leeuwarden, The Netherlands) for 4–8 h until cloudy before transferring to 50 mL of nutrient broth (Fluka 70122, St. Louis, MO, USA). After two days, cultures were transferred to a Leighton–Doi broth. Cultures were monitored daily until more than 95% of spores were observed via microscopy (Western Digital PMD-1 USB2 1.09, software v 2.0.0, Westover Scientific, Mill Creek, WA, USA). Cultures were then centrifuged (30 min, 10,000 g, 4 °C). Pellets were rinsed once with cold diH_2_O, centrifuged, and then resuspended in 10 mL of diH_2_O. One milliliter aliquots were heat-treated before spore concentration was determined via plate counting.

### 2.2. Conjugate Preparation

The capture antibody (rabbit anti-*B. anthracis* IgG, cat# TC-7009-002, Tetracore Inc., Rockville, MD, USA) was conjugated to magnetic beads (Dynabeads MyOne Tosylactivated cat# 65501, Life Technologies, Oslo, Norway) according to the manufacturer’s instructions. Prior to conjugation, the antibody was buffer-exchanged into the appropriate conjugation buffer using centrifugal filter devices (Amicon Ultra, cat# UFC505096, EMD Millipore, Billerica, MA, USA). Four monoclonal detection antibodies (cat# C86702M and C86910M, Meridian Life Science Inc., Memphis, TN, USA and cat# TC-7016-002 and TC-7018-002, Tetracore Inc.) were conjugated separately to glucose oxidase using Lightning-Link Glucose Oxidase Conjugation kits (cat# 706-0010, Novus Biologicals LLC, Littleton, CO, USA). Briefly, the capture antibody was conjugated to magnetic beads in a 0.1 M sodium borate buffer, pH 9.5, for 24 h at 37 °C. Detection antibodies were conjugated to glucose oxidase in a 50 mM sodium phosphate buffer, pH 7.5, for 24 h at room temperature. Conjugates were stored in a PBS buffer, pH 7.4, with 0.1% NaN_3_ at 4 °C.

### 2.3. Sample Preparation

A master mix consisted of 50 ng/sample of each of the four mAb-GOX conjugates and 10 μg/sample polyclonal Ab-magnetic bead conjugate in an incubation buffer (0.1 M MOPS, pH 7.0, 0.2 M NaCl, 1% BSA, and 0.1% Tween 20). Spore sample aliquots (100 μL) were incubated with a master mix (100 μL) in pre-lubricated microcentrifuge tubes (Costar 3207) by inversion (Labquake, Thermo Scientific, Waltham, MA, USA) for 30 min at room temperature. Tubes were then placed on a magnetic tube holder (cat# 101414-700), 1.0 mL of incubation buffer was added to each without disturbing the pellets, and the holder was inverted several times. After 2 min, the sample supernatant was removed, and the wash procedure was repeated twice more with 1.0 mL of wash buffer (0.1 M MOPS, pH 7.0, 0.05% Tween 20). Washed magnetic bead pellets were stored at 4 °C for up to 24 h before being tested.

*Bacillus* spore stocks were serially diluted in 0.1% Tween 20 to obtain desired testing concentrations. Viability was confirmed by plate counting (colony forming units). To generate a standard curve all sample concentrations were prepared and coded for PHAD (Portable Hazardous Agent Detector) operators (blinded samples). For environmental testing, 990 μL of a test condition (see Table 1) or negative control sample (0.1% Tween 20 only) was spiked with 10 μL of *B. anthracis* spores with 10^7^ cfu/mL dilution for 10^5^ cfu/mL spore testing concentration. Sample pellets were prepared as described above. For soil testing, differing amounts of soil were added to 10 mL of 0.1% Tween 20 and vortexed for 30 s, and 990 μL of supernatant was then immediately removed and spiked with 10 μL of *B. anthracis* spores with 10^7^ cfu/mL dilution. Soil samples were prepared as before except the BSA concentration in the incubation buffer was increased to 5% (w/v). For each condition tested, an equal number of pure culture samples were tested for comparison on the same day to determine % inhibition.

### 2.4. Amperometric Testing

**Figure 1:**
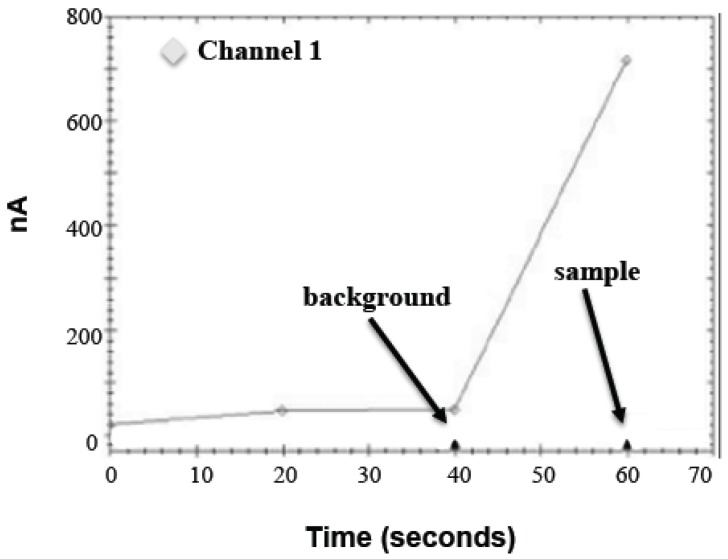
Amperogram (screenshot) of a typical PHAD (Portable Hazardous Agent Detector) instrument run. All the data presented here was obtained while the PHAD instrument was in single-channel mode, i.e., Channel 1 in the sample above.

The sample bead pellet was resuspended in a 50 μL substrate solution (1.75 mM ABTS, 1.75 U/mL HRP, and 88 mM D-glucose in 0.1 M MOPS, pH 7.0), inverted for 5 min at room temperature, placed on a magnetic tube rack, and immediately analyzed on the PHAD instrument as shown in Figure 1. The software records three current measurements at 20 s intervals over one minute. A blank substrate (20 and 40 s measurements) and a sample substrate (60 s measurement) are added to the electrode 5 s before the end of each interval. Background (40 s measurement) is subtracted from the sample measurement to determine net signal. Electrodes were reused intraday and washed once with the 50 μL substrate buffer between runs up to approximately 30 times a day with no noticeable drifting (data not shown).

### 2.5. Instrumentation

The PHAD instrument consists of a 127 mm × 85 mm × 42 mm injection-molded ABS/PC (acrylonitrile butadiene styrene/polycarbonate) case containing a single high-density, 6-layer printed circuit board (PCB) populated with surface mount components on both sides. The PCB schematic, artwork, and manufacturing documentation was created with PCB Artist V2.0 layout software (Advanced Circuits, Aurora, CO, USA). A PIC18f4550 microcomputer chip running embedded software from its internal flash memory performs instrument control, communication and digital processing. The PHAD instrument is connected to a laptop via USB, which supplies the device with an operational power of 5 V. Alternatively, power can be supplied using two AA batteries. The instrument, which has eight input channels, digitizes the input current signal (16-bit resolution), adds a time-stamp, and transmits the data to the host workstation via USB interface. The conversion is scaled for a measurement range of 0–4 μA and a 0.1 nA resolution. A 0–2 V (with a 1 mV resolution) direct current bias voltage is applied to each input (the default 0.4 V bias was used for this work). This voltage is controlled in real time in response to host workstation USB commands.

### 2.6. Software

Software development consisted of two phases: the development of embedded software within the PIC18F4550 microcomputer onboard the PHAD instrument and the development of a workstation simulator application on a desktop computer running Windows 7. The workstation simulator application was necessary to complete the development and testing of the PHAD-embedded software operation and the USB communications. Embedded software was created and debugged using an MPLAB ICD 3 in-circuit development interface, MPLAB IDE V8.91 software, and an MPLAB C18 compiler (all from Microchip Technology Inc., Chandler, AZ, USA). The embedded software was created using C language. The workstation simulator was developed for debugging of real-time operations and USB communications and was created on a Windows 7 computer using a C# compiler running under Microsoft Visual Studio 2012 to generate and display real-time USB communications to and from the instrument. Workstation software (v1.0.0.7) was developed using dotNet 4.0 using Microsoft Visual Studio 2010 (Microsoft Corp., Redmond, WA, USA) and compiled to run on Windows XP or Windows 7. The workstation allows the user to create, save, and execute scripts to be sent to the instrument. Resulting data is then visualized and can be saved to disk for further analysis. USB communication is based on a Generic HID USB Communication library (waitingforfriday.com). Visualization makes use of the ZedGraph open source charting libraries.

### 2.7. Electrodes

The electrodes used with the PHAD instrument were made of 50 nm pure gold sputtered onto a 10 mm Toray Lumirror S10 polyester (Materion Corp, Mayfield Heights, OH, USA) backboard. The solder mask (FOC-800, Taiyo America Inc., Carson City, NV, USA) application, laser ablation, and sheet cutting was performed by Conductive Technologies Inc. (York, PA, USA). A Plexiglas well (Proto Labs Inc., Maple Plain, MN, USA) is attached via a double-sided adhesive to form the electrochemical cell. Prior to assembly, the exposed gold surfaces of the electrode were cleaned with isopropanol and deionized water. Electrodes were used dozens of times within a single day (washed with substrate buffer between samples) without noticeable drift.

## 3. Results

**Figure 2:**
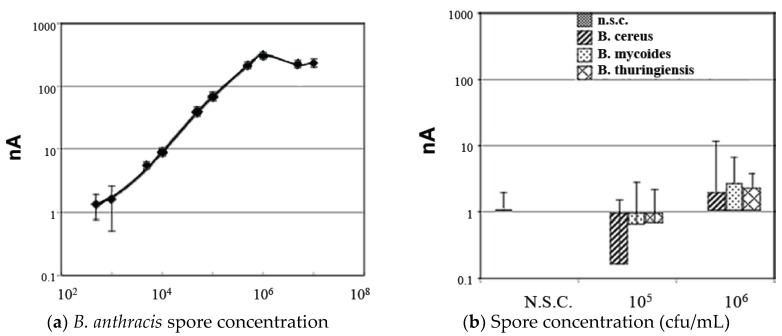
(**a**) Standard curve for detection of pure *B. anthracis* spore suspensions (spores serially diluted in 0.1% Tween 20). Each data point represents at least 10 blindly tested sample replicates. Error bars = +/− standard deviation (SD). (**b**) Negative control samples including no spore controls (0.1% Tween 20 without spores) and spores of other *Bacillus* species tested in the assay at the indicated concentrations.

The immunomagnetic separation (IMS) procedure used has been previously developed [21]. Figure 2a shows a representative standard curve for pure culture suspensions of *B. anthracis* spores over a wide range of blinded spore concentrations. Signal variability (error bars) was minimal for samples greater than 1000 spores noted.

To evaluate the specificity of the method for detecting *B. anthracis* spores, high concentrations of spores from related *Bacillus* species *B. cereus*, *B. Mycoides*, and *B. thuringiensis* were also tested in the assay as shown in Figure 2b. At both 10^5^ and 10^6^ cfu/mL concentrations, the mean signal (n ≥ 10) for each of these test species remained below the limit of detection (LOD), demonstrating a good degree of specificity for the assay, although some slight cross-reactivity with these related species was noticed at concentrations of 10^6^ cfu/mL and higher.

Robustness testing of the assay was done in the presence of various potential environmental interferents as shown in Table 1. Here, *B. anthracis* spores were spiked into each environmental condition for a final concentration of 10^5^ cfu/mL. These were compared to pure culture samples at the same spore concentration in order to determine % inhibition for each condition. All test conditions contained 0.1% Tween 20 to prevent spore aggregation. A range of interferent concentrations was initially tested to determine the maximum tolerated by the assay; defined as that causing less than 20% mean inhibition compared to controls. The interferents tested included spores from other *Bacillus* species such as *B. cereus*, *B. Mycoides*, and *B. thuringiensis*, which can be found in soil along with *B. anthracis*, common inorganic salts, low and high pH conditions, phenol (which is sometimes used as a spore preservative), and extracts of potting soil and locally collected soil.

Each interferent at the indicated concentration was spiked with 10^5^ cfu/mL *B. anthracis* spores and compared to pure culture samples of the same spore concentration. Negative controls (i.e., interferents tested with no added *B. anthracis* spores) were also run for each condition and all gave signals below the LOD with the exception of 4/10 *B. mycoides* spores samples and 1/10 *B. thuringiensis* spores samples at 10^6^ cfu/mL concentration, which were slightly above the LOD (data not shown). Notably, testing of soil samples required an increased concentration of blocking agent (5% BSA) in the IMS incubation buffer to prevent false signal in the negative controls. A small subset of pure culture *B. anthracis* spores samples with 5% BSA in the incubation buffer showed negligible mean signal deviation from samples containing 1% BSA (data not shown).

The presence of spores up to 10^6^ cfu/mL of *B. cereus*, *B. Mycoides*, and *B. thuringiensis* had minimal effect on the detection of *B. anthracis* spores. Inorganic salts showed varying degrees of inhibition in the assay but a concentration of at least 50 mM was tolerated for all of the salts tested. Phenol was tolerated at up 1.0% concentration (v/v). A range of pH points from 1 to 14 was tested in the assay using citrate, phosphate, and borate buffers. Results indicate pH levels between 5.0 and 10.0 with a buffer concentration of up to 50 mM were tolerated by the assay. Testing of soil samples was done by vortexing different concentrations of soil in 0.1% Tween 20 for 30 s, and supernatant was then removed, spiked with *B. anthracis* spores at a concentration of 10^5^ cfu/mL, and compared with control samples as before to determine % inhibition. The pH of soil extracts tested ranged from 5.5 to 8.1. The concentration of the BSA blocking agent in the IMS incubation buffer was increased to 5% to prevent false positive signals from occurring in negative control samples (samples run without the addition of spores). With this modification, the assay was able to tolerate potting soil and locally collected soil extracts at concentrations of up to 1% (w/v).

## 4. Discussion and Conclusions

The PHAD assay was developed to be a fast and sensitive method for anthrax spore detection under field conditions. As done previously, the capture and detection antibody conjugates were incubated with anthrax spores in a single step to reduce assay time compared to other sandwich immunoassays [21]. Tween 20 in the assay minimizes spore aggregation thereby maximizing spore surface area available for antibody binding. The signal was generated by glucose oxidase (coupled with anthrax spores via antibody binding), oxidizing glucose and generating hydrogen peroxide in the process. HRP, in the presence of ABTS, reduces hydrogen peroxide to water and forms an ABTS radical cation [14,15]. Compared to the previously used DCPIP substrate system, this ABTS method has improved the signal-to-noise ratio and reduced the time for signal development to a 5 min incubation at room temperature prior to amperometric measurement. In addition, buffer conditions for IMS and spore dilutions were modified for compatibility with environmental samples. Soil spiked sample signals were higher compared to non-soil controls, presumably due to the presence of magnetic particles in the soil extracts. For these samples, the BSA concentration was increased fivefold to 5% which minimized the effect. Potting soil was include in the analysis since it tends to be less loamy than locally collected soils, hence causing greater interference. The 20% signal reduction threshold for the maximum tolerated interferent concentrations (Table 1) was considered acceptable since this was within normal immunoassay variability for detecting anthrax. Overall, compared to reported conventional ELISAs for anthrax spore detection [4,22], PHAD has improved sensitivity, robustness and/or substantially reduced run times. Nucleic acid sequence (DNA or RNA), dipicolinic acid (DPA), and surface resonance (Raman spectroscopy)-based methods of anthrax spore detection all require sample processing procedures (often including sample enrichment) and equipment limiting the portability and practicality in the field [6,23,24,25].

The second generation PHAD instrument used in this work has a 16-fold greater reportable range and 2.5-fold greater resolution and is smaller (527 cm^3^) and lighter (5.5 oz). A measurement range of 0–4 μA with a 0.1 nA resolution allowed the use of the faster developing ABTS/HRP substrate system, which would have saturated our first PHAD instrument with a reportable range of only 250 nA and a 0.25 nA resolution [21]. The first instrument necessitated 10 buffer exchanges and subsequent background adjustments (zeroing) to maximize the reportable signal output range. These steps have been eliminated with our current instrument. Enhanced software adds cyclic voltammetry capability along with other attributes. The PHAD instrument, being compact, portable, and rugged, is designed for field use. It is also designed with an onboard tube rotator and magnetic rack to minimize transport of additional equipment, though these attributes were not used in this work. For testing purposes, the PHAD instrument was used only in single-channel mode but is capable of running up to eight samples simultaneously (in tandem, similar to a plate reader).

In conclusion, this article presents results for a rapid and sensitive assay for the detection of *Bacillus anthracis* spores in pure culture or environmental samples using a combination of immunomagnetic separation and amperometric quantitation. The method uses a polyclonal anti-*B. anthracis* antibody conjugated to magnetic beads and GOX-conjugated monoclonal antibodies for the capture and detection of spores, along with the amperometric measurement of GOX activity using the electron mediator ABTS in the presence of HRP and glucose. This method is able to measure as few as 500 spores in less than one hour total assay time and has a quantitative detection range from 5 × 10^3^ to 5 × 10^6^ cfu/mL. The specificity of the assay for *B. anthracis* spores depends on the antibodies used. The antibodies used here showed minimal cross-reactivity with spores of other related *Bacillus* species, albeit the number of strains used was limited and vegetative cell preparations were not included. The performance of the assay in the presence of various possible environmental interferents including soil demonstrates its potential usefulness in the testing of field samples, thus we believe this method is a promising tool for the future detection of anthrax spores in the field.

## Figures and Tables

**Table 1:** List of the maximum tolerated concentration of potential environmental interferents and % inhibition.

Potential Interferent	Max. Conc. Tolerated ^1^	% Inhibition	Std dev (%)
*Bacillus cereus* spores	10^6^ cfu/mL	11.8	11.0 (n = 8)
*Bacillus mycoides* spores	10^6^ cfu/mL	11.1	9.1 (n = 8)
*Bacillus thuringiensis* spores	10^6^ cfu/mL	5.6	10.1 (n = 10)
Calcium Chloride	75 mM	9.0	8.2 (n = 11)
Magnesium Chloride	100 mM	9.5	6.4 (n = 11)
Magnesium Sulfate	75 mM	10.4	3.9 (n = 11)
Potassium Chloride	150 mM	14.3	6.3 (n = 9)
Sodium Chloride	150 mM	14.6	4.3 (n = 8)
Sodium Phosphate (pH 7.0)	50 mM	12.9	6.6 (n = 8)
Low pH (sodium citrate pH 5.0)	50 mM	14.9	6.1 (n = 8)
High pH (sodium borate pH 10.0)	50 mM	10.6	6.2 (n = 8)
Phenol	1.0% (v/v)	6.7	6.1 (n = 10)
Potting soil extract	1.0% (w/v)	13.1	11.7 (n = 30)
Local soil extract ^2^	1.0% (w/v)	11.0	8.0 (n = 13)

^1^ Defined as the maximum concentration of interferent causing less than 20% average inhibition of assay signal compared with control samples in water with 0.1% Tween 20. ^2^ Local soil was collected outside our laboratory in Aiea, HI, USA.
